# Downregulation of CDC20 Increases Radiosensitivity through Mcl-1/p-Chk1-Mediated DNA Damage and Apoptosis in Tumor Cells

**DOI:** 10.3390/ijms21186692

**Published:** 2020-09-12

**Authors:** Yang Gao, Pengbo Wen, Bin Chen, Guanshuo Hu, Lijun Wu, An Xu, Guoping Zhao

**Affiliations:** 1 Key Laboratory of High Magnetic Field and Ion Beam Physical Biology, Hefei Institutes of Physical Science, Chinese Academy of Sciences; Anhui Province Key Laboratory of Environmental Toxicology and Pollution Control Technology, Hefei 230031, China; gy.sunny@foxmail.com (Y.G.); upets@mail.ustc.edu.cn (B.C.); hgs12345@mail.ustc.edu.cn (G.H.); anxu@ipp.ac.cn (A.X.); ljw@ipp.ac.cn (L.W.); 2 School of Graduate Students, University of Science and Technology of China, Hefei 230026, China; 3 Department of Bioinformatics, School of Medical Informatics, Xuzhou Medical University, Xuzhou 221004, China; wenpb@mail.ustc.edu.cn

**Keywords:** CDC20, radiotherapy, Mcl-1, p-Chk1, mitochondrial-dependent apoptotic pathway

## Abstract

Radiotherapy is an important modality for the local control of human cancers, but the radioresistance induced by aberrant apoptotic signaling is a hallmark of cancers. Restoring the aberrant apoptotic pathways is an emerging strategy for cancer radiotherapy. In this study, we determined that targeting cell division cycle 20 (CDC20) radiosensitized colorectal cancer (CRC) cells through mitochondrial-dependent apoptotic signaling. CDC20 was overexpressed in CRC cells and upregulated after radiation. Inhibiting CDC20 activities genetically or pharmacologically suppressed the proliferation and increased radiation-induced DNA damage and intrinsic apoptosis in CRC cells. Mechanistically, knockdown of CDC20 suppressed the expression of antiapoptotic protein Mcl-1 but not other Bcl-2 family proteins. The expressions of CDC20 and Mcl-1 respond to radiation simultaneously through direct interaction, as evidenced by immunoprecipitation and glutathione S-transferase (GST) pull-down assays. Subsequently, decreased Mcl-1 expression inhibited the expression level of phosphorylated checkpoint kinase 1 (p-Chk1), thereby resulting in impaired DNA damage repair through downregulating the homologous recombination repair protein Rad51 and finally causing apoptotic signaling. In addition, both CDC20 and Chk1 inhibitors together, through in vivo studies, confirmed the radiosensitizing effect of CDC20 via inhibiting Mcl-1 and p-Chk1 expression. In summary, our results indicate that targeting CDC20 is a promising strategy to improve cancer radiotherapy.

## 1. Introduction

Radiation therapy is one of the three main approaches to cancer treatment. Factors such as radiation type/dose, radiosensitivity/radioresistance, clinical stage of cancer, and age of the patient can affect the outcome of radiotherapy [1]. However, human cancers are increasingly recognized as a heterogeneous disease, and not all patients derive a survival benefit from radiotherapy. Radiation resistance can easily lead to incomplete cure, recurrence, and metastasis [2]. Therefore, understanding the mechanism contributing to radioresistance is pivotal to exploring strategies to improve cancer radiotherapy.

Cell division cycle 20 (CDC20), a homolog of *Saccharomyces cerevisiae* cell division cycle 20 protein, is a target molecule in the cell-cycle checkpoint [3]. As a coactivator of APC (anaphase-promoting complex/cyclosome, also called APC/C), CDC20 regulates cell-cycle progression by forming an E3 ubiquitin ligase subcomplex with APC [4]. Studies showed that APC^Cdc20^ exerts its function in mitosis by targeting a variety of key cell-cycle regulators including securin and cyclin B for ubiquitin-mediated destruction [5,6]. Accumulating evidence indicates that CDC20 plays an important role in the development of human tumors [7]. CDC20 is overexpressed in a variety of human tumors including colorectal cancer (CRC) [8,9]. For example, CDC20 is overexpressed in both CRC cell lines and primary cancer tissues compared to normal colorectal epithelial cells and paracancerous tissue samples. It is noteworthy that CDC20 expression is associated with clinical stage, metastasis, and short overall survival, suggesting that CDC20 may be an independent biomarker for predicting the prognosis of human CRC [10]. The initial role of CDC20 was elucidated primarily in regulating cell-cycle progression [7]. However, studies showed that CDC20 is also involved in apoptotic signaling. Manchado et al. found that depletion of CDC20 or inhibition of APC may lead to increased apoptosis by inhibiting Pttg1/securin [11]. CDC20 governs apoptosis by mediating the degradation of phosphatidylcholine-specific phospholipase C in hepatocellular carcinoma cells [12]. These studies indicate that CDC20 is involved not only in the regulation of the cell cycle but also apoptotic signaling. However, the detailed mechanism is unknown, and it is unclear whether CDC20 participates in the radiation response or affects CRC cell radiosensitivity.

As the first antiapoptotic protein in the Bcl-2 family, Mcl-1 is involved in the apoptosis, differentiation, and cell-cycle regulation of various cancer cells, and it is essential for cancer cell survival and growth [13,14]. Previous studies found a close relationship between Mcl-1 and tumor chemotherapy resistance [15,16]. Targeting both Mcl-1 and ATM was reported to increase cisplatin sensitivity in non-small-cell lung cancer [17]. miR-30 can increase the apoptosis of hematopoietic cells under radiation exposure by targeting Mcl-1 [18]. Harley et al. found that Mcl-1 is a substrate of CDC20, which can induce apoptosis by promoting the degradation of the antiapoptotic protein Mcl-1 [19].

The genome surveillance mechanism responds to radiation-induced DNA damage processes that cause a delay or arrest in progression of the cell cycle at the boundary of G1/S phases or G2/M phases [20]. Checkpoint kinase 1 (Chk1), the core of the genomic surveillance pathway, is induced by DNA damage, which then transduces checkpoint signals and promotes cell-cycle arrest and DNA damage repair [21]. Depending on the degree of repair, the repair process can result in either cell survival or apoptosis [22]. Notably, after DNA damage and Chk1/Chk2 activation, Mcl-1 downregulates Chk1 through ATR serine/threonine kinase as a retro-control step [23,24].

In this study, we mainly detected the effect of CDC20 on apoptotic signaling pathways in CRC cells after radiation. We clarified that knockdown of CDC20 restrained proliferation and DNA damage repair and increased radiation-induced intrinsic apoptosis in CRC cells. Moreover, attenuated CDC20 expression decreased the expression of Mcl-1 and then inhibited p-Chk1, thus leading to impaired DNA damage repair and induced cell apoptosis. These results provide convincing evidence that the CDC20/Mcl-1/p-Chk1 pathway is a promising target sensitive to cancer radiotherapy.

## 2. Results

### 2.1. CDC20 Is Overexpressed in Human Cancer Cells and Upregulated after Radiation

**Figure 1:**
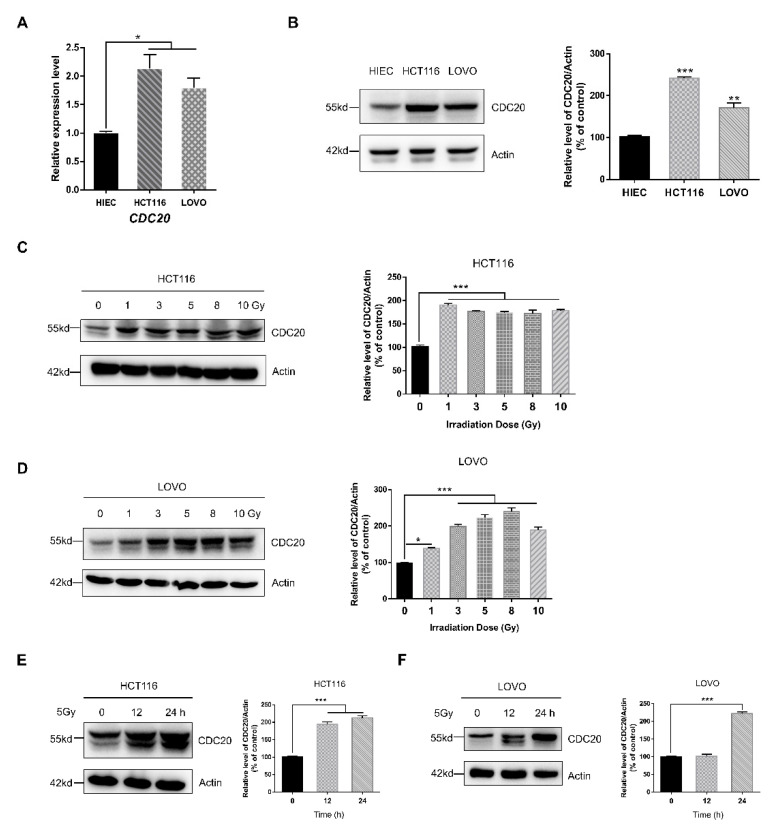
Cell division cycle 20 (CDC20) was overexpressed in human cancer cells and upregulated after radiation. (**A**,**B**) Transcriptional (**A**) and translational levels (**B**) of CDC20 in HIEC cells, HCT116 cells, and LOVO cells were detected by quantitative real-time PCR and immunoblotting. (**C**,**D**) HCT116 cells (**C**) and LOVO cells (**D**) were irradiated with different doses of γ-radiation, and the protein levels of CDC20 were analyzed by Western blotting 24 h later. (**E**,**F**) The expression of CDC20 in HCT116 cells (**E**) and LOVO cells (**F**) at different time points after 5 Gy γ-radiation. Actin was used as an internal control. Data were pooled from three independent experiments, and the results are represented as mean ± SD; * *p* < 0.05, ** *p* < 0.01, *** *p* < 0.001.

It was reported that CDC20 is significantly overexpressed in different cancer types [8]. In order to better understand the role of CDC20 in cancer, we obtained RNA-Seq-based CDC20 expression levels in 33 human cancers from The Cancer Genome Atlas (TCGA) rich dataset. Scatter plots of messenger RNA (mRNA) levels show that the majority of CDC20 is expressed at varying levels of moderate to high in patient samples (Figure S1A, Supplementary Materials). We noticed that CDC20 expression in colorectal cancer (CRC) is especially higher, and increased CDC20 expression is closely related to the clinicopathological progression of CRC (Figure S1B,C, Supplementary Materials). To confirm the results of the TCGA data, CDC20 expression in two CRC cells lines (HCT116 and LOVO) and in the normal human intestinal epithelial cell line (HIEC) was analyzed. The expression level of CDC20 was higher in colorectal cells, particularly in HCT116 cells (Figure 1A,B). Although we found that CDC20 is overexpressed in CRC cells, it is unclear whether CDC20 participates in radiation response or affects CRC cell radiosensitivity. Therefore, we investigated the effects of gamma-ray radiation on CDC20 expression in HCT116 and LOVO cells. As shown in Figure 1C,D, 24 h following exposure, the expression levels of CDC20 were significantly increased in both HCT116 and LOVO cells after 1–10 Gy radiation. The protein levels of CDC20 were also upregulated after 12 or 24 h following 5 Gy of radiation (Figure 1E,F). In addition, we also examined the response of CDC20 to radiation in HIEC cells. As shown in Figure S1D,E, although the expression of CDC20 was low in HIEC cells, radiation still upregulated its expression. Taken together, those above results showed that, compared with normal cells, CDC20 was particularly overexpressed in CRC and was upregulated after radiation, highlighting its potential as a novel therapeutic target for advanced CRC.

### 2.2. Knockdown of CDC20 Sensitizes CRC Cells to Radiation through Stimulating DNA Damage and Intrinsic Apoptotic Pathway

**Figure 2:**
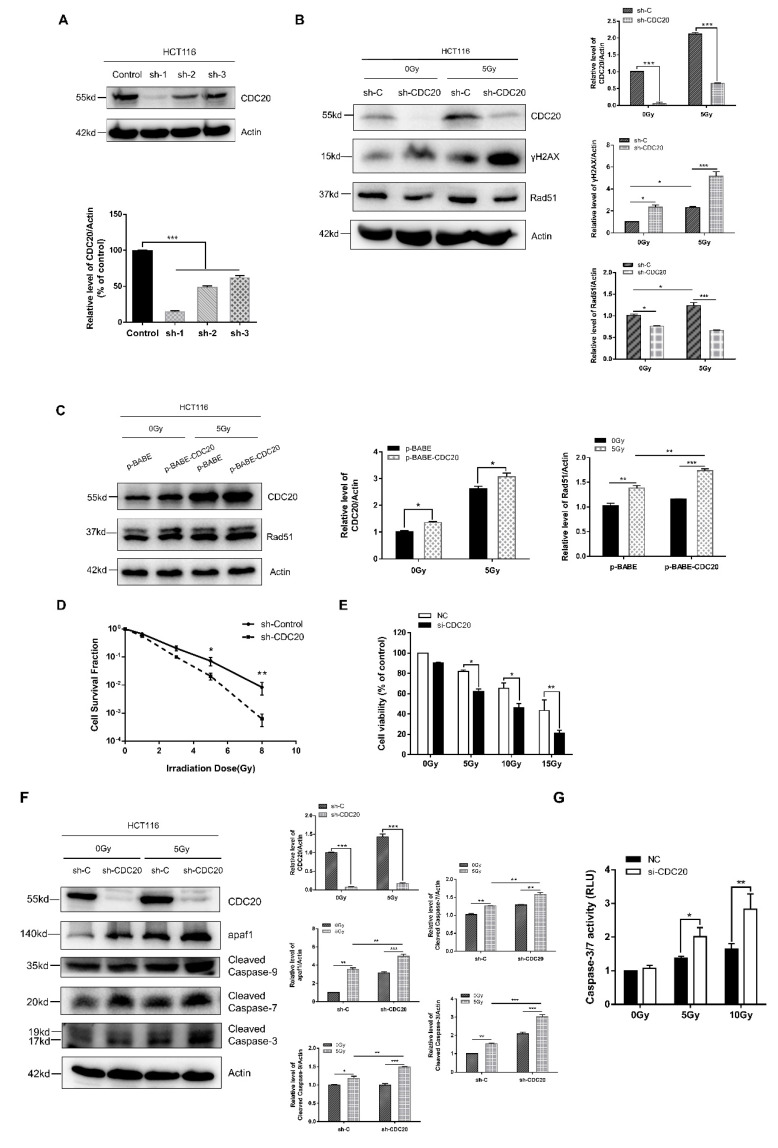

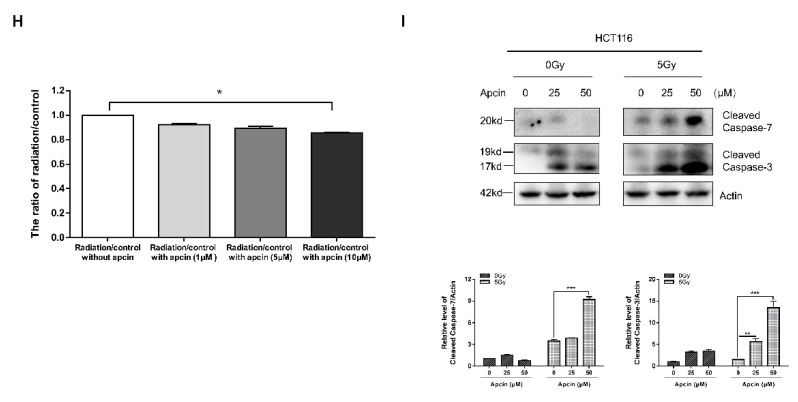
CDC20 attenuates the sensitivity of HCT116 cells to gamma-rays. (**A**) CDC20 was stably reduced in HCT116 cells by lentiviral infection. The expression levels of CDC20 were determined by immunoblotting analysis (sh-1/2/3 stands for three short hairpin RNA segments). (**B**) The expression levels of γH2AX and Rad51 were detected by Western blotting in control and CDC20 knockdown cells at 24 h after 5 Gy gamma-ray irradiation. (**C**) The expression level of Rad51 was detected in control and CDC20 overexpression cells at 24 h after 5 Gy gamma-ray irradiation. (**D**) A clongenic assay of HCT116 cells with reduced CDC20 expression and control vector cells was carried out after irradiation with different doses of gamma-rays. (**E**) HCT116 cells with reduced CDC20 expression and control vector cells were exposed with the indicated doses of gamma-ray irradiation, and cell viability was measured 24 h later. (**F**,**G**) Higher expression level of apaf1, cleaved caspase-9, cleaved caspase-7, and cleaved caspase-3 (**F**) and more robust caspase-3/7 activation (**G**) were detected in CDC20 knockdown HCT116 cells compared with control vector cells 24 h after 5 Gy gamma-ray irradiation. (**H**) HCT116 cells were pretreated with different doses of apcin (0–10 µM) for 24 h before 5 Gy gamma-ray irradiation, and cell viability was detected 24 h after irradiation. (**I**) Cells were preincubated with apcin (25 or 50 µM) for 24 h before 5 Gy gamma-ray irradiation; then, the expression levels of cleaved caspase-7 and cleaved caspase-3 were determined by Western blot. Actin was used as an internal control. Data were pooled from three independent experiments and the results are represented as mean ± SD; * *p* < 0.05, ** *p* < 0.01, *** *p* < 0.001.

To elucidate the role of CDC20 in radiation, we examined whether CDC20 is essential to mediate the DNA damage response and apoptotic signaling of radiation. As shown in Figure 2A, CDC20 expression was stably reduced in HCT116 cells using short hairpin RNA (shRNA). Histone H2AX phosphorylation on a serine four residues from the carboxyl terminus (producing γH2AX) is a sensitive and robust biomarker for DNA damage, the expression level of γH2AX was significantly increased in CDC20 knockdown cells compared with control cells after radiation (Figure 2B). Moreover, the expression level of Rad51, a key protein in the homologous recombination repair pathway, was markedly reduced in CDC20 knockdown cells and upregulated in CDC20 overexpression cells compared with control cells after radiation (Figure 2B,C). These data suggested that suppression of CDC20 enhanced radiation-induced DNA damage through inhibiting Rad51 in CRC cells.

Next, a clonogenic assay together with 3-4,5-dimethyl-2-thiazolyl-2,5-diphenyl-2-H-tetrazolium bromide (MTT) assay indicated that knockdown of CDC20 results in decreased cell viability after radiation (Figure 2D,E). Consistently, the higher expression levels of cleaved caspase-9, cleaved caspase-7, cleaved caspase-3, and caspase-3/7 activation were detected in CDC20 knockdown cells compared with control vector cells upon radiation (Figure 2F,G). In particular, it was noticed that the expression level of apaf1 was significantly increased after CDC20 knockdown together with radiation treatment compared with radiation treatment or CDC20 knockdown alone (Figure 2F), indicating that the intrinsic apoptotic pathway induced by radiation treatment in CDC20 knockdown cells was triggered by apaf1. Furthermore, apcin, as a specific CDC20 inhibitor, was used to further evaluate the role of CDC20 in radiosensitivity. Apcin is a specific inhibitor with direct action against the APC^Cdc20^ complex, which occupies the D-box binding pocket within the WD40 domain to block the connection between CDC20 and APC, thereby regulating the activity of its downstream substrates and exerting its biological functions [25,26]. As shown in Figure 2H, apcin (10 μM) pretreatment plus gamma-ray radiation had significantly greater inhibitory effects on cell viability than radiation exposure alone. Moreover, the expression levels of cleaved caspase-7 and cleaved caspase-3 induced by radiation were significantly increased in the apcin pretreated group compared to the non-pretreated group (Figure 2I). These results indicate that the downregulation of CDC20 increases the radiosensitivity of CRC cells mainly by promoting the radiation-induced apoptosis pathway.

To complement the knockdown experiments, we performed CDC20 overexpression studies in HCT116 cells. We noted that ectopic expression of CDC20 led to an enhancement of cell proliferation (Figure S2A, Supplementary Materials). Furthermore, the expression levels of cleaved caspase-3, cleaved caspase-7, and γH2AX were decreased in CDC20-overexpressing cells compared with control vector cells upon radiation (Figure S2B, Supplementary Materials). These data are consistent with the effects of reducing CDC20 expression (Figure 2D–G), further validating the radioresistant role of CDC20 in CRC cells.

### 2.3. Antiapoptotic Protein Mcl-1 Is a CDC20-Interacting Protein

**Figure 3:**
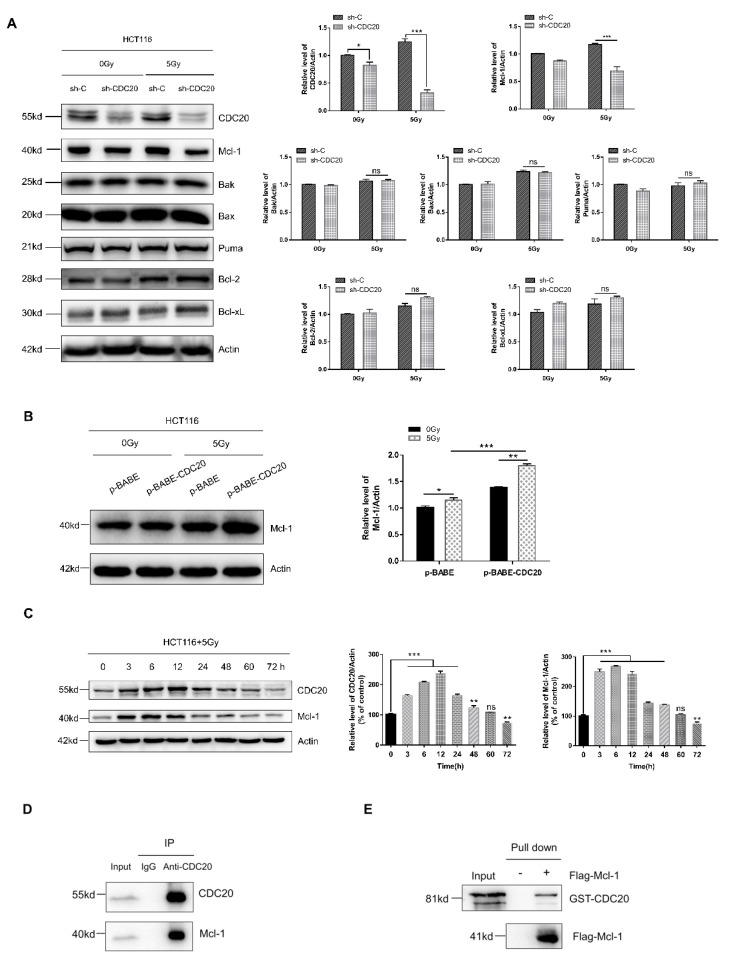
CDC20 inhibits radiation-induced apoptosis signals by interacting with Mcl-1. (**A**) The expression levels of Mcl-1, Bak, Bax, Puma, Bcl-2, and Bcl-xL were detected by immunoblotting analysis in CDC20 knockdown HCT116 cells compared with control vector cells 24 h after 5 Gy gamma-ray irradiation. (**B**) The expression level of Mcl-1 was determined by immunoblotting analysis in CDC20-overexpressing HCT116 cells compared with control vector cells 24 h after 5 Gy gamma-ray irradiation. (**C**) After irradiating with 5 Gy gamma-rays, the expression levels of CDC20 and Mcl-1 were detected by immunoblotting analysis at different time points. (**D**) Interaction of endogenous CDC20 with Mcl-1 was examined by Co-immunoprecipitation assay in HCT116 cells. (**E**) Purified Flag-Mcl-1 immobilized on beads was incubated with purified glutathione S-transferase (GST)-CDC20. Input and bead-bound proteins were analyzed by immunoblotting with anti-CDC20 antibody. Actin was used as an internal control. Data were pooled from three independent experiments and the results are represented as mean ± SD; * *p* < 0.05, ** *p* < 0.01, *** *p* < 0.001. ns = non significant.

Since mitochondrial-dependent apoptosis is mainly regulated by Bcl-2 family proteins [27,28], we investigated the role of Bcl-2 family proteins in CDC20-mediated radiosensitivity. As shown in Figure 3A, CDC20 knockdown cells failed to alter the expression levels of Bak, Bax, Puma, Bcl-2, and Bcl-xL compared with control vector cells upon radiation. By contrast, Mcl-1 expression was significantly decreased after CDC20 knockdown together with radiation compared with radiation alone. Consistent with CDC20 knockdown results, CDC20 overexpression combined with radiation increased the Mcl-1 expression (Figure 3B).

Next, we found that, after 5 Gy radiation treatment, the expression levels of both CDC20 and Mcl-1 proteins were increased following 0–12 h exposure and decreased following 12–72 h exposure (Figure 3C), indicating that CDC20 and Mcl-1 respond to radiation stimuli simultaneously. To further explore the role of Mcl-1 in CDC20-mediated radiosensitivity, we performed an interaction detection of endogenous CDC20 and Mcl-1 in HCT116 cells. The immunoprecipitation assay indicated a specific interaction between CDC20 and Mcl-1 protein (Figure 3D). An in vitro binding assay with purified GST-CDC20 and Flag-Mcl-1 proteins also revealed that CDC20 directly associated with Mcl-1 (Figure 3E). It should be noted that overexpressing or reducing CDC20 failed to affect the mRNA level of Mcl-1 (Figure S3A, Supplementary Materials), whereas overexpressing or reducing Mcl-1 did not affect the mRNA level of CDC20 (Figure S3B). The above results indicate that CDC20 regulates the expression of Mcl-1 through direct interaction at the translational level but not the transcriptional level.

### 2.4. Mcl-1 Increases Chk1 Phosphorylation to Regulate CDC20-Mediated DNA Damage and Apoptosis in Radiation

**Figure 4:**
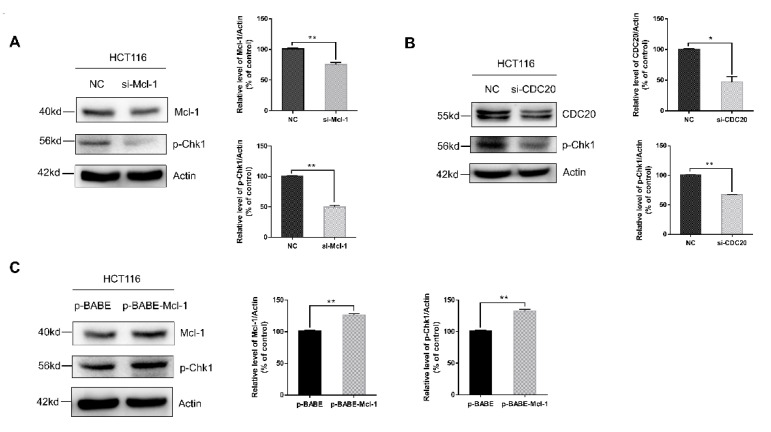

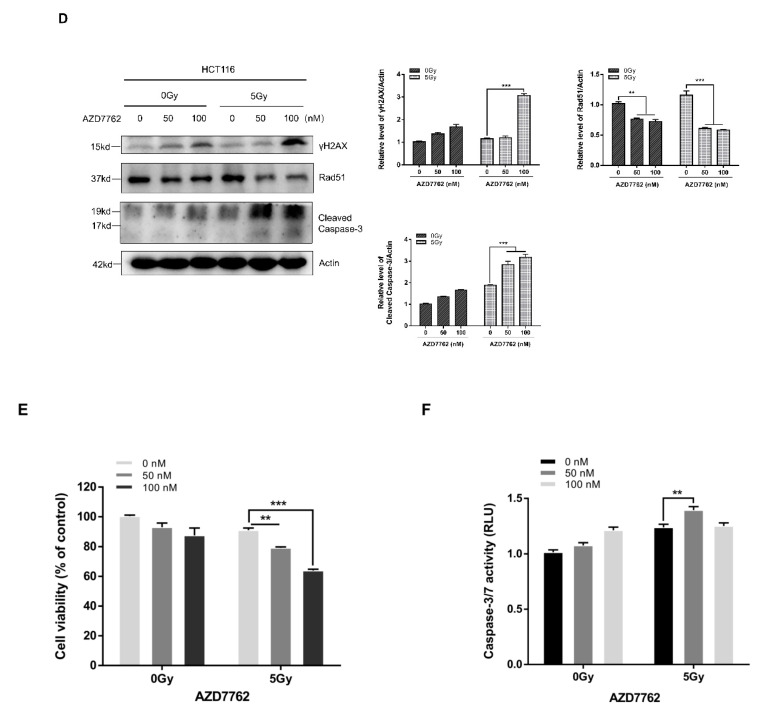
The radioresistant function of CDC20 was mediated by the Mcl-1/p-Chk1 signal axis. (**A**–**C**) The expression level of p-Chk1 was detected in Mcl-1 knockdown HCT116 cells (**A**), CDC20 knockdown HCT116 cells (**B**), and Mcl-1-overexpressing HCT116 cells (**C**). (**D**) HCT116 cells were pretreated with different doses of AZD7762 (0–100 nM) for 24 h before 5 Gy gamma-ray irradiation, and the expression levels of γH2AX, Rad51, and cleaved caspase-3 were detected 24 h after irradiation. (**E**,**F**) HCT116 cells were pretreated with different doses of AZD7762 (0–100 nM) for 24 h before 5 Gy irradiation; cell viability (**E**) and caspase-3/7 activity (**F**) were detected 24 h after 5 Gy γ-radiation. Actin was used as an internal control. Data were pooled from three independent experiments and the results are represented as mean ± SD; * *p* < 0.05, ** *p* < 0.01, *** *p* < 0.001.

Chk1 is a DNA damage checkpoint kinase that is involved in DNA damage repair in the form of phosphorylation, and its phosphorylation is indirectly regulated by Mcl-1 [23]. To investigate the role of Chk1 in CDC20-mediated radiosensitivity, we detected p-Chk1 expression in HCT116 cells with reduced or increased CDC20 or Mcl-1 expression. As shown in Figure 4A–C, reducing Mcl-1 or CDC20 decreased the expression level of p-Chk1 and overexpression of Mcl-1 increased the expression level of p-Chk1 in HCT116 cells. In order to further elucidate the role of p-Chk1 in radiosensitivity, a novel selective Chk1 and Chk2 inhibitor 1-(2-((*S*)-piperidin-3-ylcarbamoyl)-5-(3-fluorophenyl) thiophen-3-yl) urea (AZD7762) was used [29,30]. Studies showed that Chk1 inhibitor AZD7762 can significantly enhance the cytotoxic effects of gemcitabine, cisplatin, and paclitaxel [31,32]. As shown in Figure 4D, the combination of 100 nM AZD7762 pretreatment and radiation significantly increased the phosphorylation level of H2AX and decreased Rad51 protein expression compared with radiation alone. Furthermore, the expression levels of cleaved caspase-3 and caspase-3/7 activation induced by radiation were significantly increased after the treatment of AZD7762 (Figure 4D,E). The MTT assay indicated that AZD7762 (50–100 nM) pretreatment plus gamma-ray irradiation had significantly greater inhibitory effects on cell viability than radiation exposure alone (Figure 4F). These results indicated that Mcl-1 could regulate the phosphorylation of Chk1, while inhibition of p-Chk1 caused radiosensitization of CRC cells through inducing DNA damage and apoptosis.

### 2.5. Reducing CDC20 Expression Enhanced the Radiosensitivity of CRC Xenografts by Inducing Apoptosis in Tumors

**Figure 5:**
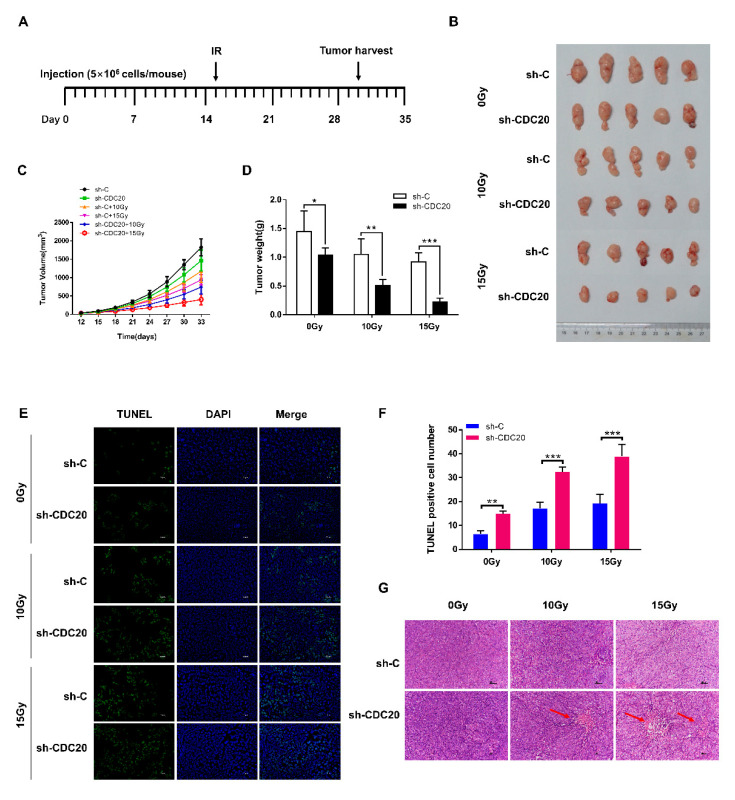

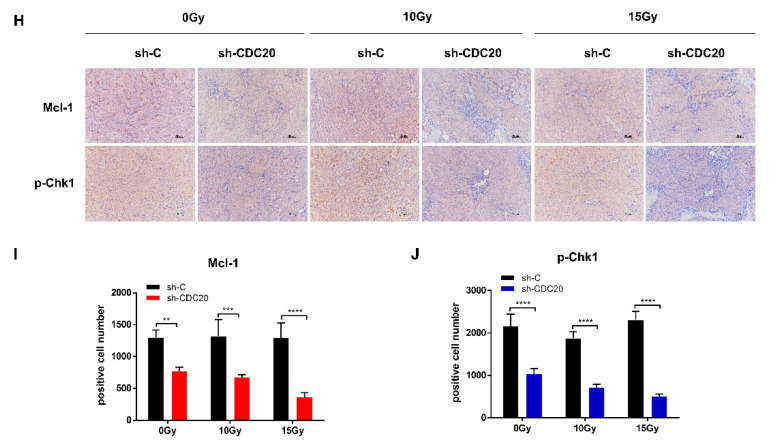
CDC20 knockdown suppresses the tumor formation of colorectal cancer (CRC) through inducing apoptosis in vivo. (**A**) Plan of animal experiments. (**B**) Tumor xenografts of each group are shown. Each group of mice was composed of five BALB/c-nu/nu mice. HCT116 control vector cells (sh-C, 5 × 10^6^) and CDC20 knockdown HCT116 cells (sh-CDC20, 5 × 10^6^) were inoculated under the dorsal skin of BALB/c-nu/nu mice. After 15 days of injection, mice were irradiated with 0 Gy, 10 Gy or 15 Gy gamma-rays. On the 33rd day after injection, the mice were sacrificed, and tumors were obtained. (**C**,**D**) The tumor volume (**C**) and net weights (**D**) of each group are shown. Tumor size was measured with dull-edged vernier calipers every 3 days, calculated as follows: volume = (length × width^2^)/2. The weight of the tumor was determined when the mice were sacrificed. (**E**) Apoptotic cells in tumor sections were identified by Terminal dexynucleotidyl Transferase (TdT)-mediated dUTP nick end labeling (TUNEL) assay. Representative images of tumor sections from sh-C and sh-CDC20 mice under different doses of gamma-ray treatment are shown. The scale bar represents 25 µm. (**F**) The number of apoptotic cells shown in (**E**) was quantified using GraphPad Prism. (**G**) Necrotic cells in tumor sections were identified by Hematoxylin-Eosin (H&E) staining. Representative images of tumor sections from sh-C and sh-CDC20 mice under different doses of gamma-ray treatment are shown. The scale bar represents 50 µm. (**H**) Immunohistochemical analysis of Mcl-1 and p-Chk1 expression in the tumor xenografts. Representative immunohistochemical staining of both Mcl-1 and p-Chk1 from sh-C and sh-CDC20 mice under different doses of gamma-ray treatment are shown (magnification, ×200). The scale bar represents 50 µm. (**I**,**J**) The number of positive cells shown in (**H**) was quantified using GraphPad Prism. Data were pooled from three independent experiments and the results are represented as mean ± SD; * *p* < 0.05, ** *p* < 0.01, *** *p* < 0.001, **** *p* < 0.0001.

To determine whether downregulation of CDC20 can sensitize CRC to radiation in vivo, wild type (WT) HCT116 and HCT116 with reduced CDC20 expression were inoculated into athymic nude mice. As shown in Figure 5A–D, reducing CDC20 expression significantly increased the inhibitory effects of gamma-rays (10 Gy or 15 Gy) on HCT116 tumor growth. The weights and volumes of tumors from the CDC20 shRNA synergized with radiation were obviously lower compared to the other group, indicating that CDC20 knockdown increases the radiosensitivity of CRC in vivo.

To examine whether the radioresistant effect of CDC20 in vivo was the result of apoptosis, we performed Terminal dexynucleotidyl Transferase (TdT)-mediated dUTP nick end labeling (TUNEL) assays in tumor tissues. As shown in Figure 5E,F, the apoptotic cell population in CDC20 shRNA synergized with radiation-treated xenografts was significantly greater than that in xenografts treated with radiation alone, indicating that radiation synergized with CDC20 shRNA enhances radiation-induced apoptosis in HCT116 xenografts. Hematoxylin-Eosin (H&E) staining results showed that CDC20 shRNA and radiation combined xenografts had poor proliferation and antiapoptotic ability (Figure 5G). Moreover, the expression levels of proteins Mcl-1 and p-Chk1 in the tumor tissues were analyzed by immunohistochemistry (Figure 5H). As shown in Figure 5I,J, the expression levels of Mcl-1 and p-Chk1 in CDC20 shRNA synergistic with the radiation-treated xenografts were significantly lower than those in xenografts treated with radiation alone. These results indicated that CDC20 knockdown improved the radiosensitivity of CRC in vivo through inhibiting Mcl-1 and p-Chk1 expression, which is consistent with the in vitro data described above.

## 3. Discussion

The role of CDC20 as an oncogene has been increasingly recognized and widely reported in a variety of human cancers [33,34]. TCGA transcriptome data indicate that CDC20 is highly expressed in human cancers (Figure S1, Supplementary Materials). CDC20 is mainly involved in regulating the cell cycle and plays an oncogenic role in human tumorigenesis [7]. CDC20 mediates the resistance to docetaxel in castration-resistant prostate cancer in a Bim-dependent manner [33]. Targeting CDC20 is a better cancer therapeutic strategy, because knocking down CDC20 can lead to mitotic arrest and cell death, and can effectively kill apoptosis-resistant cancer cells [34]. In this study, we found that radiation upregulated the expression of CDC20, and CDC20 increased the proliferation and radiation-induced DNA damage repair and inhibited intrinsic apoptosis of CRC cells (Figure 1C–F and Figure 2). Using the immunodeficient athymic nude mice, we further observed that inhibiting CDC20 activities increased the sensitivity of CRC cells to radiation by inducing apoptosis in tumors (Figure 5E–G). Collectively, our data suggested that CDC20 significantly inhibited the radiosensitivity of CRC both in vitro and in vivo.

Apoptosis is considered to be the main mechanism of radiation-induced cell death, and aberrant apoptotic signaling leads to radioresistance of most, perhaps all, types of human cancers [35]. The Bcl-2 proteins are a family of structurally related proteins that serve as central regulators of apoptosis [36]. Bcl-2 proteins can be classified into antiapoptotic (Bcl-2, Bcl-xL, Mcl-1, etc.) and proapoptotic (Bak, Bax, Puma, Bim, etc.) groups [37]. Here, we found that Mcl-1 but not other Bcl-2 family proteins were involved in CDC20-mediated apoptosis of radiation (Figure 3A). Mcl-1 is a typical antiapoptotic protein in the Bcl-2 family which regulates mitochondrial-dependent apoptosis by inhibiting proapoptotic protein function and then affecting radiosensitivity [15,38]. As shown in Figure 3A and Figure 5H, the Mcl-1 protein was downregulated by CDC20 knockdown combined with radiation in vitro and in vivo. When CDC20 was overexpressed and accompanied by radiation, the Mcl-1 expression was increased (Figure 3B). Mechanistically, the direct interaction between the CDC20 protein and Mcl-1 protein confirmed the positive correlation between CDC20 and Mcl-1 expression. CDC20 can interact directly with Mcl-1 using immunoprecipitation and GST pull-down assays (Figure 3D,E). Those above results suggest that CDC20 may increase the activity of Mcl-1 by enhancing the stability of Mcl-1 protein, thereby blocking the apoptotic signal. Furthermore, although most Bcl-2 family proteins did not change in CDC20 knockdown cells, the apoptosis cascade still occurred (Figure 2F). After radiation, apaf1, which is highly expressed in CDC20 knockdown cells, may increase the possibility of physical interaction with procaspase-9 molecules and activate them. Activated caspase-9 activates the downstream effector proteases caspases-3 and caspases-7 that perform cell death programs. In short, CDC20 knockdown can promote the expression of apaf1, which triggers the downstream apoptosis cascade and finally induces mitochondrial-dependent intrinsic apoptosis, thereby enhancing the radiosensitization effect of CRC cells.

Radiation-induced DNA damage can disrupt homeostasis, causing DNA damage repair or apoptosis [20]. Genome surveillance mechanisms can ensure that DNA damage checkpoints are activated to arrest cell-cycle progression in order to repair damaged chromosomes [39]. As a core component of the genome surveillance mechanism, Chk1 activates the trigger of the G2 checkpoint during DNA damage repair [40,41]. In this study, we found that knockdown of CDC20 downregulated the expression level of p-Chk1 in vitro and in vivo (Figure 4B and Figure 5H). Furthermore, CDC20 shRNA synergistic with the radiation-treated xenografts decreased p-Chk1 expression compared to the radiation alone group (Figure 5H,J). Moreover, Chk1 inhibitor, AZD7762, inhibited phosphorylation of Chk1 and increased radiation-induced apoptotic signaling (Figure 4D). These corroborative results indicate that Chk1 functions as an important regulator in response to radiation stimuli. Studies showed that transient transfection of Mcl-1 results in increased expression of phosphorylated ser345 Chk1 and accumulation of cells in the G2 phase. Knockdown of Mcl-1 abolished Chk1 phosphorylation in response to DNA damage [23]. In CRC cells, we found that reducing/increasing Mcl-1 protein decreased/increased the expression level of p-Chk1 (Figure 4A,C). On the basis of the above results, we postulate that CDC20 interacted directly with antiapoptotic protein Mcl-1 to regulate Mcl-1 expression after radiation, which increased the phosphorylation of Chk1 and finally caused radiation resistance. In this study, CDC20 was also involved in radiation-induced DNA damage response. As shown in Figure 2B,C, reducing/increasing CDC20 protein decreased/increased the expression level of Rad51. In addition, the expression level of γH2AX was significantly upregulated by CDC20 knockdown. As an inhibitor of Chk1, AZD7762 decreased Rad51 expression and increased γH2AX expression after radiation (Figure 4D). These results further suggested that knockdown of CDC20 decreased the expression level of Rad51 through inhibiting the p-Chk1, thereby aggravating DNA damage. Moreover, we found that protease inhibitor MG132 increased the expression level of Rad51 (Figure S4, Supplementary Materials), suggesting that CDC20 regulated the degradation of Rad51 protein by affecting its ubiquitination.

**Figure 6:**
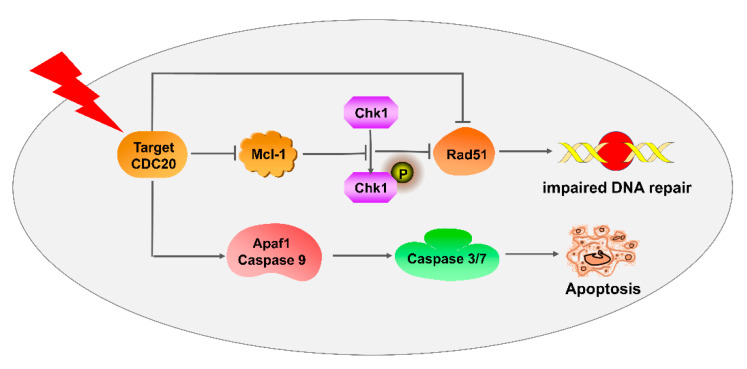
Schematic representation of the mechanism of CDC20-mediated radiosensitization of CRC cells.

In summary, this study reveals that inhibiting CDC20 activity genetically or pharmacologically restrains DNA damage repair and enhances radiation-induced intrinsic apoptosis of CRC in vitro and in vivo. Knockdown of CDC20 decreases the activity of the antiapoptotic protein Mcl-1 and then inhibits the phosphorylation of checkpoint kinase Chk1, thus suppressing DNA homologous recombination repair protein Rad51 and inducing cell apoptosis to cause radiosensitivity (Figure 6). Given its potential role in resistance to radiation, the CDC20/Mcl-1/p-Chk1 pathway might be a promising target to overcome radioresistance and increase the efficacy of cancer therapy.

## 4. Materials and Methods

### 4.1. Cell Culure and Irradiaion

The human colon cancer cell line HCT116, HEK293T cell line, and human small intestine epithelium cell line (HIEC) were kindly provided by Dr. Chi Li (University of Louisville, Louisville, Kentucky) and were cultured in Dulbecco’s modified Eagle’s medium (HyClone, Logan, UT, USA). Human colon cancer cell line LOVO was purchased from the Cell Bank of the Chinese Academy of Sciences (Shanghai, China) and was cultured in DMEM/F12 (HyClone, Logan, UT, USA). All cell lines were supplemented with 10% fetal bovine serum (Biological Industries, Beit HaEmek, Israel) and 1% penicillin/streptomycin (Beyotime, Shanghai, China) at 37 °C with 5% CO_2_.

Irradiation was carried out in a gamma-ray irradiator (Biobeam GM gamma irradiator, Leipzig, Germany). Cells were irradiated by high-energy gamma-rays, with a dose rate of 3.27 Gy/min, automatically controlled by the computer. The equipment is maintained and calibrated every year by the manufacturer to ensure the precision of radiation dose.

### 4.2. Reagents

MTT (3-4,5-dimethyl-2-thiazolyl-2,5-diphenyl-2-H-tetrazolium bromide) was purchased from Sigma-Aldrich (Merck KGaA, Darmstadt, Germany). Trizol reagent was purchased from Invitrogen (Shanghai, China). The Transcript^®^one-step gDNA removal and complementary DNA (cDNA) synthesis supermix kit was purchased from TransGen Biotech (Beijing, China). SuperReal PreMix (SYBR GREEN) was purchased from Qiagen (Shanghai, China). Primers for quantitative real-time PCR were purchased from GENEWIZ (Suzhou, China). The Chk1 inhibitor AZD7762 was purchased from Selleck (Houston, TX, USA). The CDC20 inhibitor apcin was purchased from Tocris (Minneapolis, MN, USA). Dimethyl sulfoxide (DMSO) and other chemicals were purchased from Sangon (Shanghai, China). Protein G Sepharose beads was purchased from Beyotime (Shanghai, China).

### 4.3. Cell Viability Assay

Cell viability was measured using the MTT assay. Briefly, the treated cells were cultured in a 96-well plate; then, after incubation for 24 h at 37 °C, 5 µL of MTT reagent (5 mg/mL in PBS) was added to each well, followed by incubation for 4 h. After that, the cells were incubated with 150 µL of DMSO for 30 min to dissolve the crystals. The absorbance of each well at 490 nm was determined using a Multimode Reader of SpectraMax M2 (Molecular Devices, Sunnyvale, CA, USA).

### 4.4. Colony Formation Assay

A total of 800 cells were seeded in a 60 mm dish. After irradiation, the dishes were incubated for two weeks at 37 °C in a 5% CO_2_ incubator for 20 days. Then, the dishes were washed with PBS, fixed with a solution containing methanol/acetic acid (*v*/*v* = 9:1) for 30 min, and subsequently stained with crystal violet for 30 min. The colonies containing more than 50 cells per colony were scored and plotted.

### 4.5. Caspase Assays

The Caspase-3/7 activity assay kit was purchased from Promega (Madison, WI, USA) and used as previously described [42]. In this assay, the proluminescent substrate containing the amino-acid sequence Asp–Glu–Val–Asp (DEVD) is cleaved by activated caspase 3/7, leading to the release of a luciferase substrate (aminoluciferin) and the generation of a luminescent signal. Data were presented as relative luminescent units (RLU), which were normalized to the corresponding values of control cells as an indicator of caspase-3/7 activities.

### 4.6. Transfection of Small Interfering RNA (siRNA) and shRNA Sequence

The siRNA (si-CDC20, si-Mcl-1) and shRNA sequences were purchased from GenePharma (Shanghai, China) and Genewiz (Suzhou, China), and siRNA transfection was carried out using Lipofectamine 2000 (Thermo Fishier, Carlsbad, CA, USA) according to the manufacturer’s protocols. The siRNA sequences and shRNA sequences are listed in Tables S1 and S2 (Supplementary Materials)..

### 4.7. Lentivirus Production and Cell Culture

To produce the lentivirus, HEK293T cells were transfected with the shRNA plasmids along with the helper plasmids psPAX2 and pMD2.G, with PEI (Thermo Fishier, Carlsbad, CA, USA) transfection reagent used as a lipid transport milieu. Lentivirus in the medium was obtained 48–72 h after transfection. We infected cells using respective lentiviral supernatants with 10 µg/mL polybrene (Sigma, Merck KGaA, Darmstadt, Germany) to generate HCT116 cells with reduced CDC20 expression or the vector control. Stable cell lines were obtained by culturing cells in the medium containing 1.5 mg/mL puromycin (Beyotime, Shanghai, China). Cells were all cultured in a 5% CO_2_-humidified incubator at 37 °C.

### 4.8. Quantitative Real-Time PCR

Total RNA was prepared from cultured cells using Trizol according to the manufacturer’s protocol. The kit used for reverse-transcription PCR (RT-PCR) was that previously mentioned. Amplification of the generated cDNA was carried out in SYBR Green Realtime PCR Master Mix with the ABI StepOne^TM^ Real-Time PCR instrument. Each measurement was performed in triplicate, and the results were normalized by the expression of the U6 gene. Fold change relative to mean value was determined using the 2^−△△Ct^ method. The primers used for qRT-PCR analysis are listed in Table S3 (Supplementary Materials). Reaction parameters were as follows: 95 °C for 2 min, 95 °C for 5 s, and 60 °C for 10 s, for 40 cycles.

### 4.9. Western Blot Analysis

Western blot was conducted as previously described [35]. The antibodies for Western blotting were as follows: Mcl-1 (#5453, 1:1000, Cell Signaling Technology, Danvers, MA, USA), p-Chk1 (#2348, 1:1000, Cell Signaling Technology, Danvers, MA, USA), caspase-7 (#9492S, 1:1000, Cell Signaling Technology, Danvers, MA, USA), Bak (#3814, 1:1000, Cell Signaling Technology, Danvers, MA, USA), γH2AX (#9718, 1:1000, Cell Signaling Technology, Danvers, MA, USA), CDC20 (10252-1-AP, 1:1000, Proteintech, Rosemont, IL, USA), Bax (50599-2-Ig, 1:1000, Proteintech, Rosemont, IL, USA), Puma (55120-1-Ap, 1:1000, Proteintech, Rosemont, IL, USA), Bcl-2 (12789-1-Ap, 1:1000, Proteintech, Rosemont, IL, USA), Bcl-xL (10783-1-Ap, 1:1000, Proteintech, Rosemont, IL, USA), cleaved caspase-3 (MAB835, 1:1000, R&D Systems, Minneapolis, MN, USA), cleaved caspase-9 (sc8355, 1:1000, Santa Cruz, CA, USA), Rad51 (ab133534, 1:1000, Abcam, Cambridge, UK), apaf1(ab234436, 1:1000, Abcam, Cambridge, UK) and β-actin (T0022,1:3000, Affinity, San Francisco, CA, USA).

### 4.10. Immunoprecipitation Assay

Total cell lysates were prepared using RIPA Lysis Buffer (Beyotime, Shanghai, China) containing protease inhibitors. After centrifugation, the primary antibody was added to the supernatant and incubated at 4 °C for 12 h while gently stirring. Protein G Sepharose Bead Slurry was then added to capture the protein complex. After incubation at 4 °C for 3 h with gentle agitation, the samples were centrifuged for 5 min at 4 °C. The supernatant was discarded and the pellet was washed with RIPA Lysis Buffer. Finally, the immunoprecipitates were resuspended for Western blot analysis using SDS-PAGE loading buffer.

### 4.11. Protein Expression and Purification

Protein purification was carried out as described previously [43]. DNA sequences encoding CDC20 were cloned into the pGEX-6P-1 vector. The construct was transformed into *Escherichia coli* Rosetta2 (DE3) cells. The cells were cultured at 37 °C until the A_600_ reached 0.6 and then induced with 0.2 mM isopropyl β-d-thiogalactoside (Takara, Shiga, Japan) for 16 h at 25 °C. The cells were suspended in 50 mM Tris-HCl (pH 8.0) containing 50 mM NaCl, 1 mM Dithiothreitol (DTT), and 1 mg/mL lysozyme, incubated on ice for 30 min, and sonicated. After spinning at 8000× *g* for 15 min at 4 °C, the supernatant was incubated with glutathione Sepharose beads (GE Healthcare, Shanghai, China) for 12 h. Reduced glutathione (Beyotime, Shanghai, China) was then used to wash the agarose beads and collect the eluent for the indicated experiments. To purify Flag-Mcl-1 proteins, a Flag-Mcl-1 expressing construct was transfected into HEK293T cells. Cell lysates were immunoprecipitated with anti-Flag M2 affinity beads (Sigma-Aldrich, Merck KGaA, Darmstadt, Germany).

### 4.12. Xenograft Studies of Nude Mice

Four- to six-week-old male BALB/c-nu/nu mice were randomly divided into six groups, each containing five mice, and then stably transfected tumor cells (5 × 10^6^) were inoculated subcutaneously in the right dorsal flank of each mouse. After 15 days of injection, the treated mice were irradiated with 0 Gy, 10 Gy, or 15 Gy gamma-rays. Tumor dimensions and volumes (mm^3^) were measured and calculated with calipers every three days. In the end, the nude mice were sacrificed by cervical dislocation in the third week after exposure to irradiation, and the tumors were harvested. The tumor tissues were fixed in 4% paraformaldehyde to obtain sections for the TUNEL assay, H&E staining, and immunohistochemical analysis. All animal experimental procedures were approved by the Institutional Animal Care and Use Committee of Anhui Medical University (3 June 2019, LLSC2019031).

### 4.13. Immunohistochemistry Analysis

Harvested tumors were fixed in 10% neutral buffered formalin for 48 h, embedded in paraffin blocks, and sectioned at 5 µm onto slides. Sections were immersed in boiling sodium citrate buffer (pH 6.0) for antigen retrieval. Next, sections were incubated with 3% H_2_O_2_ for 10 min to block endogenous peroxidase activity. Then, the slides were incubated with first antibodies against Mcl-1 (10252-1-AP, 1:500, Proteintech, Rosemont, IL, USA) and p-Chk1 (AF3008, 1:100 Affinity, San Francisco, CA, USA) at 4 °C overnight. Next, the slides were incubated with secondary antibody at 37 °C for 30 min. Immunoreactivity was detected using the Strept Avidin-Biotin Complex (SABC) method.

### 4.14. Statistical Analysis

All experiments were independently repeated at least three times, and all statistical data were presented as the means ± standard error. The statistical significance of differences was determined using Student’s *t*-test with GraphPad Prism 6 (GraphPad Software, Inc., San Diego, CA, USA) for comparison between two groups and ANOVA for comparison among multiple groups. Probability (*p*) values <0.05 were considered to represent statistically significant differences (* *p* < 0.05, ** *p* < 0.01, *** *p* < 0.001; ns, not significant).

## Supplementary Materials

Supplementary Materials can be found at https://www.mdpi.com/1422-0067/21/18/6692/s1.
