# Supplementary material for: Downregulation of CDC20 Increases Radiosensitivity through Mcl-1/p-Chk1-Mediated DNA Damage and Apoptosis in Tumor Cells
Source: Int J Mol Sci. 2020 Sep 12;21(18):6692. doi: 10.3390/ijms21186692 (PMC7555290; doi:10.3390/ijms21186692)
Supplement: Supplementary file 1 [file ijms-21-06692-s001.pdf]

Figure S1-1

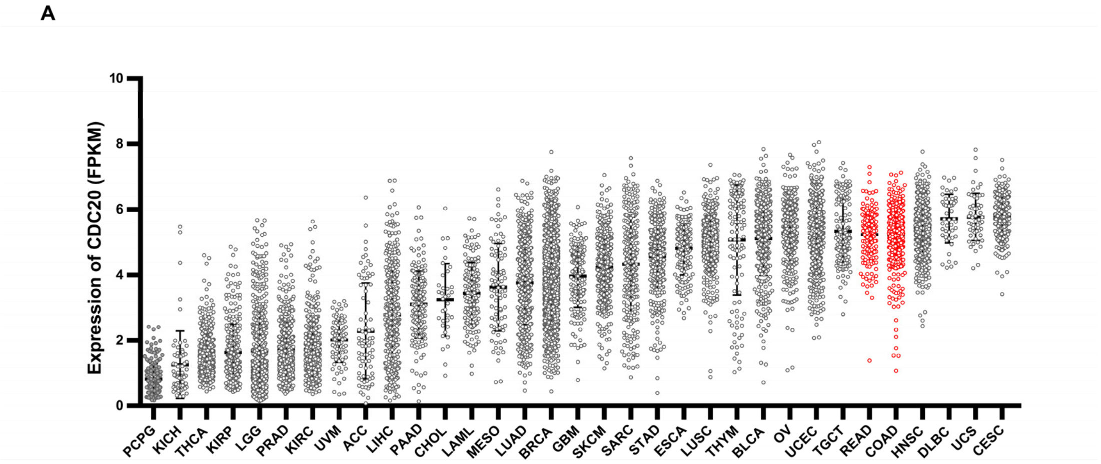

Figure S1-2

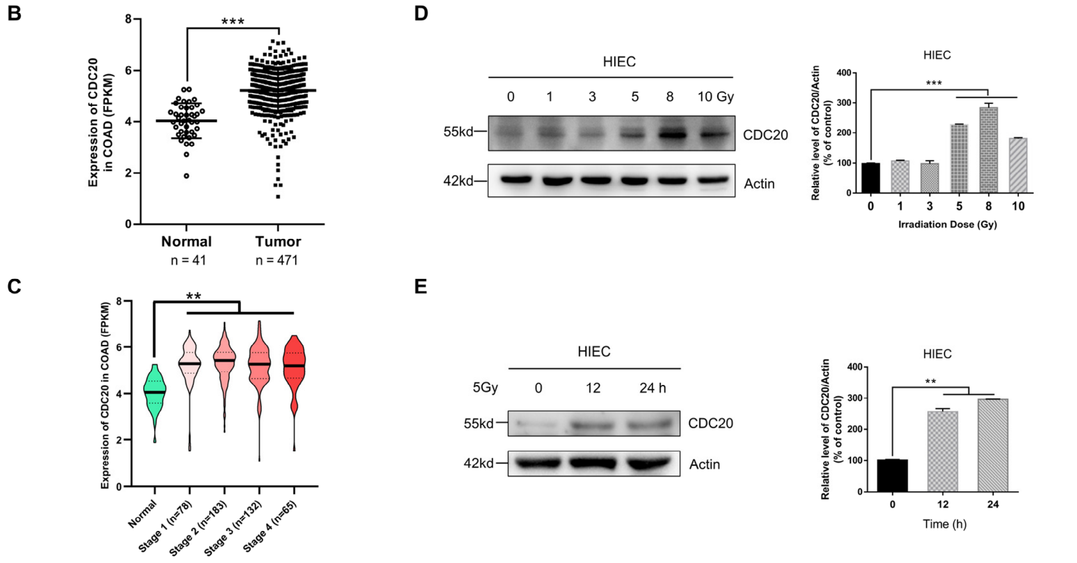

Figure S1. (A) Expression of CDC20 in 33 tumor types in the TCGA database arranged by median. **(B)** The expression level of CDC20 in CRC patients and adjacent cancer tissue samples was calculated based on the TCGA database. **(C)** Transcriptional expression of CDC20 at different stages of CRC from the TCGA database. (**D**) HIEC cells were irradiated with different doses of γ-radiation, and the protein levels of CDC20 were analyzed by western blotting 24 h later. (**E**) The expression of CDC20 in HIEC cells at different time points after 5 Gy γ-radiation. *FPKM*, Fragments per Kilobase Million; Data were pooled from three independent experiments and the results were represented as mean ± SD. ** $p < 0.01$, *** $p < 0.001$.

Figure S2

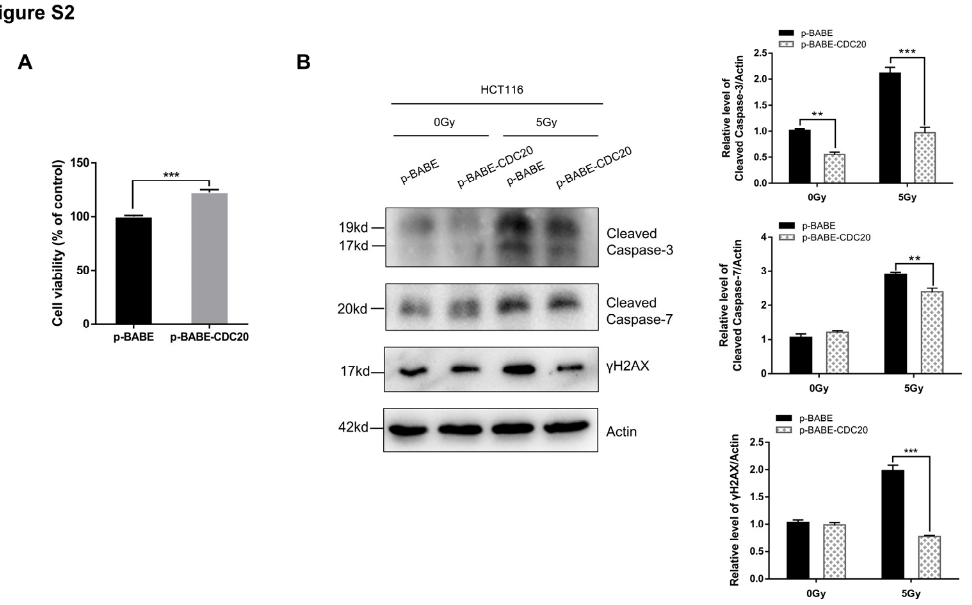

Figure S2. (A) Cell viability analysis of control and CDC20 overexpression cells. **(B)** 24 hours after 5 Gy Gamma-ray irradiations, less expression level of cleaved caspase-3, cleaved caspase-7 and γH2AX were detected in CDC20 overexpression cells compared with control vector cells. Actin was used as internal control. Data were pooled from three independent experiments and the results were represented as mean ± SD. ***p* < 0.01, ****p* < 0.001.

Figure S3

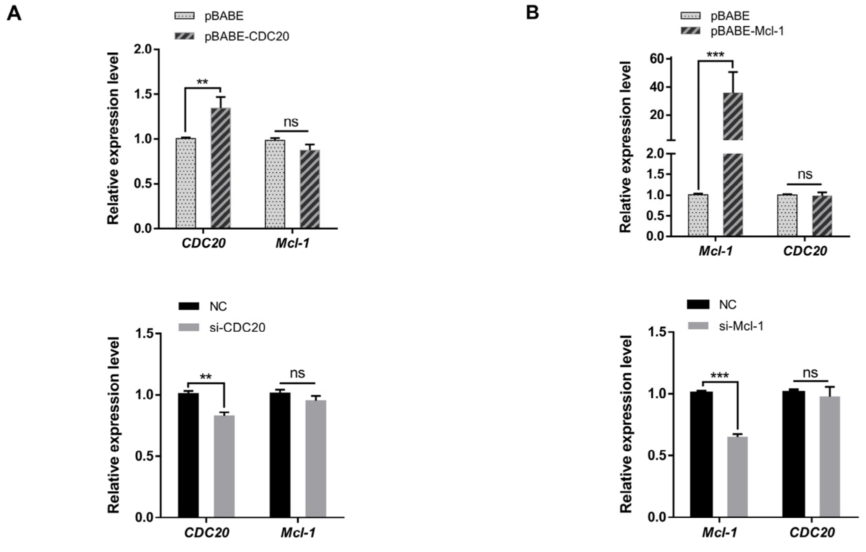

Figure S3. (A) Quantitative real-time PCR assay of Mcl-1 expression in CDC20-overexpression or CDC20 knockdown HCT116 cells compared with their corresponding control vector cells. **(B)** Quantitative real-time PCR assay of CDC20 expression in HCT116 cells Mcl-1-overexpression or Mcl-1 knockdown HCT116 cells compared with their corresponding control vector cells. Data were pooled from three independent experiments and the results were represented as mean ± SD. ***p* < 0.01, ****p* < 0.001.

Figure S4

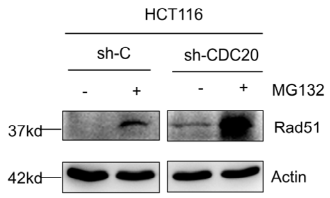

Figure S4. Immunoblotting analysis of Rad51 in CDC20 knockdown HCT116 cells and control vector cells after 4 h of treated with 10 μM MG132. Actin was used as internal control.

Table S1. siRNA sequences of siRNA-NC, siRNA-CDC20 and siRNA-Mcl-1

Gene	Forward primer	Reverse primer
NC	5'-UUCUCCGAACGUGUCACGUTT-3'	5'-ACGUGACACGUUCGGAGAATT-3'
si-CDC20	5'-GGAAGACCUGCCGUUACAUTT-3'	5'-AUGUAACGGCAGGUCUUCCTT-3'
si-Mcl-1	5'-GCUAAACACUUGAAGACCATT-3'	5'-UGGUCUUCAAGUGUUUAGCTT-3'

Table S2. shRNA sequence of CDC20

Gene	Sequence
shRNA-1	F: 5'- CCGGGCAGAAACGGCTTCGAAATATCTCGAGATATTTCGAAGCCGTTTCTGCTTTTTG-3'
	R: 5'- AATTCAAAAAGCAGAAACGGCTTCGAAATATCTCGAGATATTTCGAAGCCGTTTCTGC-3'
shRNA-2	F: 5'- CCGGATGCGCCTGAAATCCGAAATGCTCGAGCATTTCGGATTTCAGGCGCATTTTTTG-3'
	R: 5'- AATTCAAAAAATGCGCCTGAAATCCGAAATGCTCGAGCATTTCGGATTTCAGGCGCAT-3'
shRNA-3	F: 5'- CCGGCTAAGCTGGAACAGCTATATCCTCGAGGATATAGCTGTTCCAGCTTAGTTTTTG-3'
	R: 5'- AATTCAAAAACTAAGCTGGAACAGCTATATCCTCGAGGATATAGCTGTTCCAGCTTAG-3'

Table S3. Primer sequences for RT-PCR analysis

Gene	Forward primer	Reverse primer
CDC20	5’-AGACATTCACCCAGCATCAAG-3’	5’- CATCCACGGCACTCAGAC-3’

Mcl-1	5’-GTGCCTTTGTGGCTAAACACT-3’	5’-AGTCCCGTTTTGTCCTTACGA-3’
U6	5’- TCGCTTCGGCAGCACATAT -3’	5’- ATTTGCGTGTCATCCTTGC -3’
